# RvD1 Attenuated Susceptibility to Ischemic AKI in Diabetes by Downregulating Nuclear Factor-κ B Signal and Inhibiting Apoptosis

**DOI:** 10.3389/fphys.2021.651645

**Published:** 2021-07-13

**Authors:** Zheng Li, Zhiwen Liu, Hengcheng Lu, Wenni Dai, Junxiang Chen, Liyu He

**Affiliations:** Department of Nephrology, The Second Xiangya Hospital of Central South University, Changsha, China

**Keywords:** RVD1, apoptosis, NF-κ B, diabetes, ischemic kidney injury

## Abstract

**Background:**

Acute kidney injury (AKI), when occurring in diabetic kidney disease (DKD), is known to be more severe and difficult to recover from. Inflammation and apoptosis may contribute to the heightened sensitivity of, and non-recovery from, AKI in patients with DKD. Resolvin D1 (RvD1) is a potent lipid mediator which can inhibit the inflammatory response and apoptosis in many diseases. However, it has been reported that the RvD1 levels were decreased in diabetes, which may explain why DKD is more susceptible to AKI.

**Methods:**

For animal experiments, diabetic nephropathy (DN) mice were induced by streptozotocin (STZ) injection intraperitoneally. Renal ischemia–reperfusion was used to induce AKI. Blood urea nitrogen (BUN) and serum creatinine were determined using commercial kits to indicate renal function. Renal apoptosis was examined by terminal deoxynucleotidyl transferase dUTP nick-end labeling (TUNEL) assay. Real-time polymerase chain reaction (PCR) was used to detect the marker of inflammatory response. Western blot was used to detect the expression of nuclear factor-κB (NF-κB)-related proteins. For clinical study, 12 cases diagnosed with DKD were enrolled in this study, and an equal number of non-diabetic renal disease patients (NDKD) were recruited as a control group. The serum RvD1 in DKD or NDKD patients were detected through an ELISA kit.

**Results:**

In clinical study, we found that the serum RvD1 levels were decreased in DKD patients compared to those in NDKD patients. Decreased serum RvD1 levels were responsible for the susceptibility to ischemic AKI in DKD patients. In animal experiments, both the serum RvD1 and renal ALX levels were downregulated. RvD1 treatment could ameliorate renal function and histological damage after ischemic injury in DN mice. RvD1 treatment also could inhibit the inflammatory response. Di-*tert*-butyl dicarbonate (BOC-2) treatment could deteriorate renal function and histological damage after ischemic injury in non-diabetic mice. RvD1 could inhibit the NF-κB activation and suppress inflammatory response mainly by inhibiting NF-κB signaling.

**Conclusion:**

RvD1 attenuated susceptibility to ischemic AKI in diabetes by downregulating NF-κB signaling and inhibiting apoptosis. Downregulated serum RvD1 levels could be the crucial factor for susceptibility to ischemic AKI in diabetes.

## Introduction

Acute kidney injury (AKI) is a disease characterized by a decline in renal function in a short time. Many causes are responsible for AKI, including sepsis, ischemia/reperfusion (I/R), and nephrotoxins (Linkermann et al., 2014). Chronic kidney disease (CKD) is a condition induced by various causes of progressive loss of renal function in months to years (Peng et al., 2015). In developed countries, diabetes mellitus (DM) is the leading cause of CKD, which accounts for almost half of the cases of end-stage kidney disease (ESRD) (Kanwar et al., 2011). Traditionally, AKI and CKD were thought to be two unrelated syndromes. However, increasing evidence has demonstrated that AKI and CKD are interconnected (Venkatachalam et al., 2010; Chawla et al., 2014; Venkatachalam et al., 2015). On the one hand, AKI may contribute to the development and progress of CKD. On the other hand, CKD is a major risk factor for AKI (Peng et al., 2015). Peng et al. (2015) verified that diabetic nephropathy (DN) was more sensitive to ischemic injury regarding AKI to CKD. Their results revealed that renal ischemia–reperfusion induce a more severe AKI in DN models both *in vivo* and *in vitro*. Although some progress has been made in the study of the progression of AKI to CKD, the underlying mechanisms are still poorly understood. In this paper, we mainly focus on the potential mechanisms that underlie the poor prognosis of AKI in CKD.

Resolvins (RVs) are a class of anti-inflammatory bioactive small molecules with conformational specificity, which are derived from polyunsaturated fatty acids enzymatically in natural conditions (Zhao et al., 2016). Resolvin D1 (RvD1) is a potent lipid mediator able to promote inflammatory resolution in many diseases, including acute lung injury (Panera et al., 2016), insulin resistance, peritonitis, wound infection, atherosclerosis, and I/R injury (Zhao et al., 2016). In the field of nephrology, RvD1 has been verified to protect against I/R kidney injury, adriamycin-induced nephropathy, obstructive nephropathy, and kidney transplant rejection (Duffield et al., 2006; Keyes et al., 2010; Qu et al., 2012; Zhang et al., 2013). Interestingly, RvD1 serves as a renal protective factor mainly through inhibiting the inflammatory response and renal cell apoptosis (Zhao et al., 2016).

All of the data indicate that the inflammatory response and cell apoptosis may be the key targets of RvD1. In diabetes, RvD1 also could inhibit the inflammatory response and cell apoptosis in streptozotocin (STZ)-induced diabetic rats (Yin et al., 2017). However, Shi et al. (2017) confirmed that the RvD1 levels were downregulated both in the diabetes mouse models and in the high-glucose-treated retinal endothelial cells. This finding may partially explain why CKD is more sensitive to AKI. Nuclear factor (NF)-κB and nucleotide-binding oligomerization domain (NOD)-like receptor protein 3 (NLRP3) are thought to be two key regulators in inflammatory response in diabetes (Tang and Yiu, 2020). The NF-κB family is composed of five protein members: p65 (RelA), RelB, c-Rel, p50, and p52. Under normal conditions, NF-κB is blocked by its inhibitor, IKB. Persistent activation of NF-κB would promote the release of inflammatory factors and enhance the inflammatory response, finally aggravating tissue damage (Shao et al., 2016). In diabetes, endogenous danger-associated molecular patterns are generated and induce a sterile tubulointerstitial inflammatory response *via* the NF-κB signaling pathway (Tang and Yiu, 2020). The NLRP3 inflammasome links sensing of metabolic stress in the diabetic kidney to the activation of pro-inflammatory cascades *via* the induction of interleukin (IL)-1β and IL-18. Thus, the NLRP3 inflammasome plays an important role in kidney diseases through its regulation of the inflammatory response in diabetes (Tang and Yiu, 2020).

Recently, an increasing number of studies has demonstrated that RvD1 could inhibit p65 activation (an important subunit of NF-κB), which finally suppressed the NF-κB activation, resulting in a decreased inflammatory response (Zhao et al., 2016; Yin et al., 2017; Xu et al., 2018). Based on these findings, this study was initiated to assess whether RvD1 attenuated susceptibility to ischemic AKI in diabetes through inhibiting the NF-κB-mediated inflammatory response and cell apoptosis.

## Materials and Methods

### Human Patient Participants and Sample Collection

Human patients were recruited in The Second Xiangya Hospital of Central South University, and the study protocol was approved by the Ethics Committee of The Second Xiangya Hospital of Central South University (no. 2019S316). The whole-blood samples were collected with the patient’s consent, which was used for RvD1 measurement through an enzyme-linked immunosorbent assay (ELISA) kit.

The inclusion criteria were as follows: (1) diabetic kidney diseases (DKD) or non-diabetic renal disease (NDKD) patients and (2) patients with valvular heart disease or aortic dissection who need to undergo surgery with cardiopulmonary bypass and aortic cross-clamping operations. The exclusion criteria were as follows: patients with tumor, neurological diseases, and chronic kidney disease other than DKD.

In the end, 12 cases diagnosed with DKD were enrolled in this study, and an equal number of NDKD patients were recruited as a control group. The baseline characteristics of the DKD and control participants are shown in Table 2. In addition, this study is an observational one; thus, no pharmacological treatments were added. All patients underwent surgery with cardiopulmonary bypass and aortic cross-clamping operations, which were mainly done in order to simulate the renal ischemia–reperfusion environment in human patients. Patients with serum creatinine levels that increased by more than 50% relative to the baseline value 7 days after surgery were diagnosed with AKI.

**TABLE 1:** Primers used in the study.

**Variables**	**Sense primer (5′–3′)**	**Antisense primer (5′–3′)**
IL-1β	GAAATGCCACCTTTTGACAGTG	CTGGATGCTCTCATCAGGACA
IL-6	TCCAGTTGCCTTCTTGGGAC	GTACTCCAGAAGACCAGAGG
MCP-1	TAAAAACCTGGATCGGA ACCAAA	GCATTAGCTTCAGATTT ACGGGT
TNF-α	CAGGCGGTGCCTATGTCTC	CGATCACCCCGAAGTTCAGTAG
COX-2	TTCCAATCCATGTCAAAACCGT	AGTCCGGGTACAGTCACACTT
GAPDH	GGGTGTGAACCATGAGAAGT	CCAAAGTTGTCATGGATGACCT

**TABLE 2:** Baseline characteristics of DKD and NDKD (control) participants.

**Variables**	**DKD**	**NDKD**
Number	12	12
Sex (M/F)	8/4	7/5
Age (years)	49.08 ± 4.82	46.83 ± 6.37
Blood glucose (mmol/L)	8.15 ± 0.67	9.14 ± 0.17
HBA1c (%)	7.49 ± 1.04	7.99 ± 1.21
Serum creatinine (μmol/L)	195.71 ± 19.75*	64.59 ± 9.48
Blood urea nitrogen (mmol/L)	21.19 ± 7.18*	5.73 ± 2.95
24 h proteinuria (mg)	1,150.54 ± 340.78*	142.65 ± 18.32

*Values are the mean ± SD.*

M, male; F, female; HBA1C, glycosylated hemoglobin; DKD, diabetic kidney disease; NDKD, diabetic without kidney disease.

*P < 0.05 vs. *NDKD.*

### Animal Experiments

Four-week-old C57BL/6J mice were purchased from the Slaccas Animal Laboratory (Changsha, China) and housed under controlled environmental conditions (temperature of 22°C, 12 h darkness period). The protocol was approved by the Institutional Animal Care and Use Committee at Central South University (no. 2019S316). DN was induced by STZ (Sigma-Aldrich, St. Louis, MO, United States) injection intraperitoneally. For STZ induction of diabetes, mice at 4 weeks of age were injected with 50 mg/kg body weight STZ for 5 consecutive days according to a standard protocol (Breyer et al., 2005). STZ-induced mice were maintained for another 2–3 weeks before renal I/R. Animals with >250 mg/dl fasting blood glucose for two consecutive readings were considered diabetic (Yang et al., 2017). Renal I/R were performed according to a standard protocol indicated elsewhere (Wei and Dong, 2012). Briefly, mice were anesthetized with 60 mg/kg (intraperitoneally) pentobarbital sodium. Then, the kidneys of mice were exposed by bilateral flank incisions, and the renal pedicles were clamped to induce ischemia. After 30 min, the clamps were released for reperfusion. During the whole operation, the body temperature of the mice was maintained at 36.5°C. After 48 h of reperfusion, the mice were killed and the kidney tissues collected. Sham control mice underwent the same operation without renal pedicle clamping. For some groups, RvD1 (50 μg/kg; Cayman Chemical Co., Ann Arbor, MI, United States), di-*tert*-butyl dicarbonate (BOC-2, a selective RvD1 receptor inhibitor, 5 μg/kg; Santa Cruz Biotechnology, Santa Cruz, CA, United States), or TPCA-1 (5 mg/kg; APExBIO, Houston, TX, United States) was given intraperitoneally 30 min before renal I/R (Zhao et al., 2016; Pan et al., 2018).

### Quantification of RvD1

RvD1 plasma levels were measured by ELISA according to the standard protocol (Cayman Chemical Co., Ann Arbor, MI, United States), as validated in another research (Bjork et al., 2015).

### Renal Function and Apoptosis

Blood urea nitrogen (BUN) and serum creatinine were detected with a kit from BioAssay Systems (Hayward, CA, United States). Renal tubular cell apoptosis was determined by terminal deoxynucleotidyl transferase 2’-deoxyuridine, 5’-triphosphate (dUTP) nick-end labeling (TUNEL) assay using the *in situ* Cell Death Detection kit (Roche Applied Science, Indianapolis, IN, United States). For quantification, 10–20 fields were randomly selected from each tissue section and the amount of positive cells per square millimeter was evaluated.

### Morphological Studies and Immunohistochemistry

Hematoxylin and eosin (HE) staining was performed to evaluate tubulointerstitial injury severity. Immunohistochemistry analysis was adopted to assess the NLRP3 levels in paraffin sections. Sections were rehydrated and the antigens retrieved using heated citrate. Then, a primary antibody was used: NLRP3 (rabbit, 1:200; Abcam, Cambridge, MA, United States). Staining was visualized using horseradish peroxidase-coupled secondary antibodies (Vectastain Elite; Vector Laboratories, Peterborough, United Kingdom). All immunohistochemical analyses were repeated at least three times and representative images were presented. Tissue damage was examined in a blinded manner and scored by the percentage of damaged tubules: 0, no damage; 1, <25%; 2, 25–50%; 3, 50–75%; and 4, >75%.

### Extraction of Total RNA and Quantitative Real-Time Polymerase Chain Reaction

Total RNA from kidney tissues was extracted with TRIzol reagent (Thermo Fisher Scientific, Waltham, MA, United States) according to the manufacturer’s instruction. Complementary DNA was synthesized using reverse transcription reagents (TaKaRa, Dalian, China). Quantitative real-time PCR was performed with the TB Green Premix Ex Taq II reagent (TaKaRa, Dalian, China) on a LightCycler 96 Real-Time PCR System (Roche Life Science, Indianapolis, IN, United States) (Tang et al., 2019). The gene expression in each sample was analyzed in duplicate and normalized against the internal control gene glyceraldehyde 3-phosphate dehydrogenase (GAPDH). The primers used in this study are shown in Table 1.

### Western Blotting

The antibodies used in the present study were from the following sources: anti-p65 (8242), anti-p-p65 (3033), anti-GAPDH (5174), anti-IκB-α (4814), and anti-p-IκB-α (2859) from Cell Signaling Technology (Boston MA, United States). All secondary antibodies for immunoblot analysis were from Thermo Fisher Scientific (Waltham MA, United States). Kidney tissues were lysed in sodium dodecyl sulfate (SDS) sample buffer (63 mM Tris–HCl, 10% glycerol, and 2% SDS) containing protease inhibitor cocktail (Sigma-Aldrich, St. Louis, MO, United States) (Tang et al., 2019). Protein concentration was detected using bicinchoninic acid (BCA) reagent. Equal amounts of protein were loaded in each well for electrophoresis using the NuPAGE Gel System, followed by transferring onto the membranes. The membranes were incubated with the primary antibodies (dilution, 1:1,000) overnight at 4°C, then subjected to the horseradish peroxidase-conjugated secondary antibodies (dilution, 1:5,000) for 1 h at room temperature, and washed with Tris-buffered saline with Tween (TBST) three times. An enhanced chemiluminescence (ECL) kit was used to visualize the bands.

### Statistical Analysis

Data were expressed as the mean ± standard error (*SD*). A two-tailed Student’s *t*-test analyzed the statistical differences between the two groups; differences in multiple groups were determined by one-way ANOVA. *P* < 0.05 was considered significant. GraphPad Prism 5.0 (GraphPad Software Inc., San Diego, CA, United States) and SPSS 17.0 (SPSS Inc., Chicago, IL, United States) statistical software were used to analyze the data.

## Results

### Decreased Serum RvD1 Was Responsible for the Susceptibility to Ischemic AKI in DKD Patients

**FIGURE 1:**
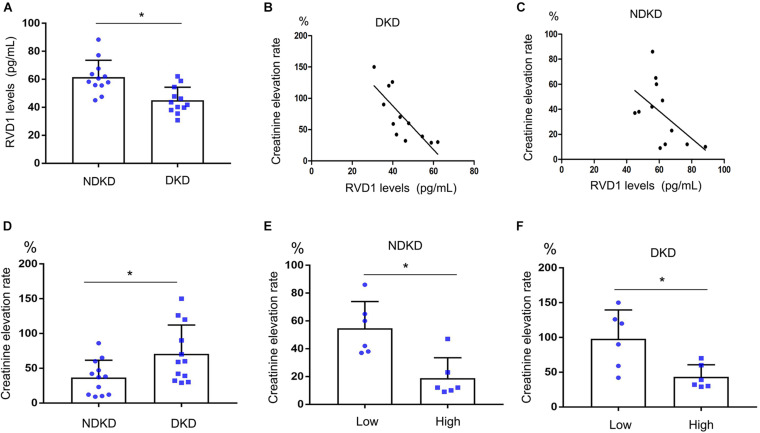
Decreased serum resolvin D1 (RvD1) was responsible for the susceptibility to ischemic acute kidney injury (AKI) in diabetic kidney disease (DKD) patients. Creatinine elevation rate: [(post-surgery serum creatinine level - pre-surgery serum creatinine level)/pre-surgery serum creatinine level] * 100%. **(A)** Serum RvD1‘**B)** Negative correlation between serum RvD1 with the creatinine elevation rate in the DKD group (*r* = -0.7928, *p* = 0.0021, Spearman’s correlation test). **(C)** Negative correlation between serum RvD1 levels with the creatinine elevation rate in the non-diabetic kidney disease (NDKD) group (*r* = -0.527, *p* = 0.078, Spearman’s correlation test). **(D)** Creatinine elevation rate in the DKD and NDKD groups. **(E,F)** Creatinine elevation rate in the NDKD and DKD subgroups. All the data are expressed as mean ± *SD*. *p < 0.05.

Firstly, we evaluated the basic characteristics of DKD and NDKD patients. Compared with the NDKD subjects, the serum creatinine, BUN, and 24 h urine protein in the DKD patients were significantly increased. There were no significant differences with respect to age, sex, blood glucose, and HBA1c (Table 2). Notably, serum RvD1 was decreased in DKD patients (Figure 1A). Besides, to facilitate the analysis of the results, we define the value of the [(post-surgery serum creatinine - pre-surgery serum creatinine)/pre-surgery serum creatinine] ^∗^ 100% as the creatinine elevation rate. Post-surgery serum creatinine was measured 7 days after surgery. Intriguingly, we found that serum RvD1 showed a good correlation with the creatinine elevation rate in the DKD group (*r* = -0.7928; Figure 1B). However, in the NDKD group, serum RvD1 showed a relatively worse correlation with the creatinine elevation rate (*r* = -0.527; Figure 1C). Besides, we also found that serum creatinine increased more relative to the baseline value in DKD patients than in NDKD patients within 7 days after surgery (Figure 1D). Then, we divided the DKD or the NDKD group into two subgroups according to the median serum RvD1. Serum RvD1 values less than the median were defined as the low group and RvD1 values more than the median were assigned to the high group (Table 3). In both the DKD and NDKD groups, within 7 days after surgery, the creatinine elevation rate increased more in the groups with low levels of RvD1 compared to that in the groups with high levels of RvD1 (Figures 1E,F). In general, within 7 days after surgery, patients with serum creatinine that increased more than 50% of the baseline could be diagnosed with AKI. As shown in Table 4, after surgery, seven patients developed AKI in the DKD group; in contrast, only three patients developed AKI in the NDKD group. Furthermore, in both the DKD and NDKD groups, low levels of RvD1 were more susceptible to ischemic AKI. In the subgroups with low levels of RvD1, AKI occurred in five patients in the DKD group and in three patients in the NDKD group. However, in the subgroups with high levels of RvD1, only two patients in the DKD group and no patient in the NDKD group developed AKI.

**TABLE 3:** Baseline characteristics of DKD and NDKD in each subgroup.

	**DKD**		**NDKD**	
	**Low**	**High**	**Low**	**High**
Number	6	6	6	6
Sex (male/female)	3/3	5/1	4/2	3/3
Age	48.83 ± 3.02	49.33 ± 6.10	47.00 ± 7.26	46.67 ± 5.34

*DKD, diabetic kidney disease; NDKD, diabetic without kidney disease.*

**TABLE 4:** Incidence of AKI after surgery.

	**RvD1 levels**	**Number**	**AKI**	**Ratio (%)**
DKD	Low	6	5	83.3
	High	6	2	33.3*
NDKD	Low	6	3	50
	High	6	0	0*

DKD, diabetic kidney disease; NDKD, diabetic without kidney disease; AKI, acute kidney injury.

*p < 0.05, compared with the corresponding low-level group.

### STZ-Induced Diabetic Mice Are More Susceptible to Ischemic AKI

**FIGURE 2:**
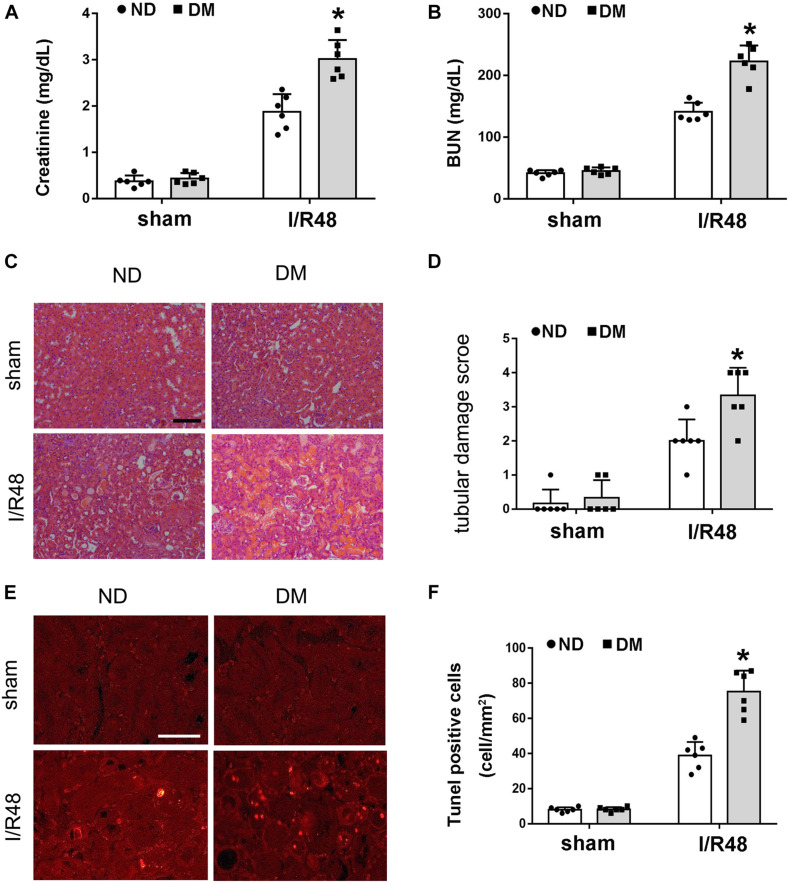
Streptozotocin (STZ)-induced diabetic mice are more susceptible to ischemic acute kidney injury (AKI). Male C57BL/6J mice were injected with STZ or vehicle solution to induce diabetic mice (DM) or non-diabetic mice (ND). The mice were then subjected to sham operation or 30 min of bilateral renal ischemia followed by 48 h of reperfusion (I/R48). **(A,B)** Serum creatinine and blood urea nitrogen (BUN). **(C)** Hematoxylin and eosin (HE) staining for renal tissues. Scale bar, 100 μm. **(D)** Semi-quantification for renal tissue damage. **(E)** TUNEL assay to reveal apoptosis of renal tissues. Scale bar, 50 μm. **(F)** Statistical analysis for TUNEL-positive cells. All the data are shown as the mean ± *SD* (*n* = 6). *p < 0.05 vs. the relevant ND group.

As described above, diabetic patients are more susceptible to AKI, resulting in a worse outcome. To verify this observation, we initially compared ischemic AKI in STZ-induced diabetic mice (DMmice) and non-diabetic mice without STZ treatment (ND mice). Functionally, 30 min of bilateral renal ischemia followed by 48 h of reperfusion (I/R48) resulted in marked increases in BUN and creatinine in both ND and DM mice. Still, the creatinine and BUN increases were substantially higher in DM mice than those in ND mice (Figures 2A,B). Renal histology analysis revealed significantly more tissue damage in DM mice after ischemic injury. I/R48 led to severe tubular damage, lysis, and necrosis (Figures 2C,D). Moreover, apoptotic cells revealed by TUNEL assay were rare in the sham control kidney tissues of both ND and DM mice. After I/R, the number of apoptotic cells increased to 74/mm^2^ cortical tissue in DM mice, but only 39/mm^2^ cortical tissue in ND mice (Figures 2E,F).

### RvD1 Attenuated Susceptibility to Ischemic AKI in Diabetes

**FIGURE 3:**
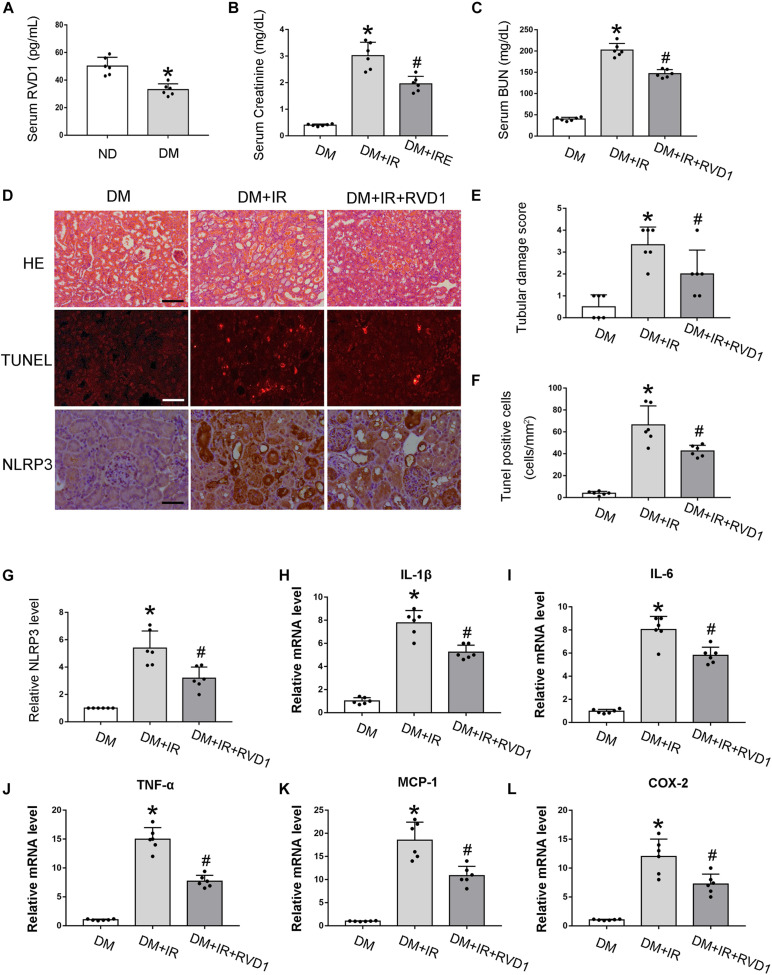
Resolvin D1 (RvD1) attenuated susceptibility to ischemic acute kidney injury (AKI) in diabetes. Male C57BL/6J mice were injected with streptozotocin (STZ) or vehicle solution to induce diabetic mice (DM) or non-diabetic mice (ND). The mice were then subjected to sham operation or 30 min of bilateral renal ischemia followed by 48 h of reperfusion (I/R48). **(A)** Serum RvD1 levels detected by ELISA. All the data are shown as the mean ± *SD* (*n* = 6). *p < 0.05 vs. the relevant ND group. **(B,C)** Serum creatinine and blood urea nitrogen (BUN). **(D)** Hematoxylin and eosin (HE) staining for renal tissues. *Scale bar*, 100 μm. TUNEL assay to reveal apoptosis of renal tissues: *scale bar*, 50 μm. Immunohistochemistry of NLRP3: scale bar, 50 μm. **(E)** Semi-quantification for renal tissue damage. **(F)** Statistical analysis for TUNEL-positive cells. **(G)** Semi-quantification for NLPR3 expression. **(H–L)** Real-time PCR for IL-1, IL-6, TNF-α, MCP-1, and COX-2. All the data in **(B,C,E–L)** are shown as the mean ± *SD* (*n* = 6). *p < 0.05 vs. the relevant DM group; ^#^*p* < 0.05 vs. the relevant DM + I/R group.

Firstly, serum RvD1 was measured by an ELISA kit according to the manufacturer’s instructions. As shown in Figure 3A, compared with the ND group, the RvD1 level in the DM group was significantly downregulated. Then, we treated DM mice with RvD1. In DM mice, 30 min of bilateral renal ischemia followed by 48 h of reperfusion resulted in marked increases in BUN and creatinine. However, RvD1 treatment could significantly ameliorate renal function when the mice suffered from ischemic injury (Figures 3B,C). HE staining revealed significantly more tissue damage in the DM + I/R group than that in the DM + I/R + RvD1 group (Figures 3D,E). RvD1 also considerably reduced the apoptotic cells when the DM mice suffered from ischemic injury (Figures 3D,F). These results indicate that RvD1 treatment could ameliorate renal function and pathological injury after ischemic injury in STZ-induced diabetic mice. Then, we also found that RvD1 treatment could inhibit the inflammatory response after ischemic injury in STZ-induced diabetic mice. Immunohistochemistry analysis showed that the NLRP3 expression was significantly increased in the kidneys of DM + I/R mice compared with DM mice, while when treated with RvD1, the expression of NLRP3 was downregulated (Figures 3D,G). Similar results were observed from real-time PCR for interleukin (IL)-1β, IL6, tumor necrosis factor (TNF)-α, MCP-1, and COX2 (Figures 3H–L). Overall, these data suggested that RvD1 attenuated susceptibility to ischemic AKI in diabetes.

### BOC-2 Treatment Could Deteriorate Renal Function and Pathological Injury After Ischemic Injury in Non-diabetic Mice

**FIGURE 4:**
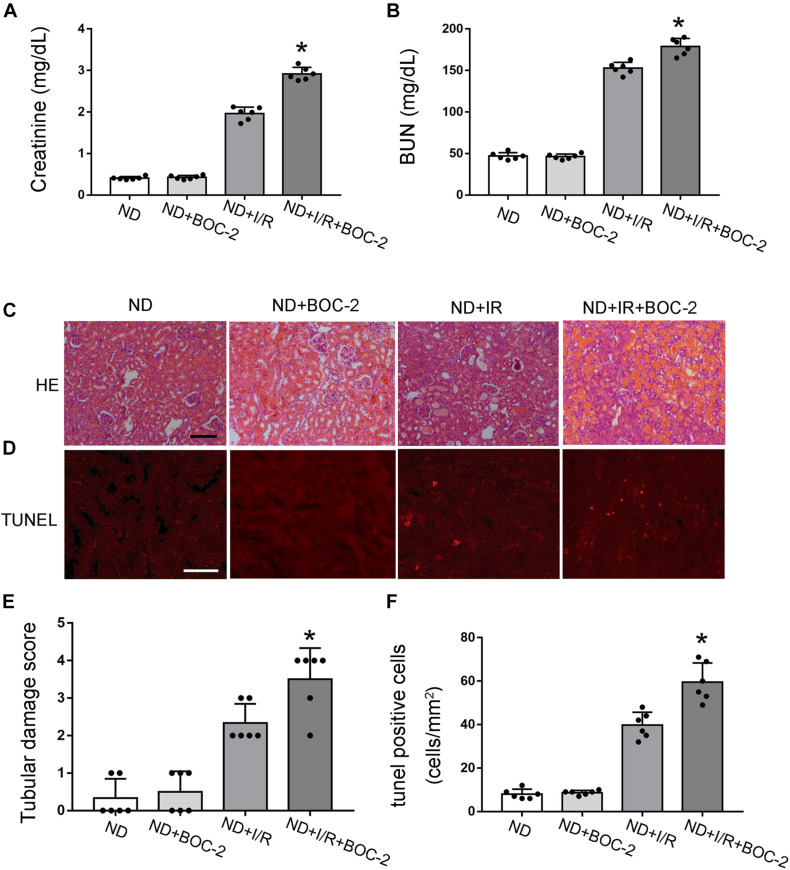
Di-*tert*-butyl dicarbonate (BOC-2) treatment could deteriorate renal function and pathological injury after ischemic injury in non-diabetic (ND) mice. Male C57BL/6J mice were subjected to sham operation or 30 min of bilateral renal ischemia followed by 48 h of reperfusion (I/R48). For the BOC-2 group, BOC-2 was given at a dose of 5 μg/kg intraperitoneally 30 min before renal ischemia–reperfusion. **(A,B)** Serum creatinine and blood urea nitrogen (BUN). **(C)** Hematoxylin and eosin (HE) staining for renal tissues. Scale bar, 100 μm. **(D)** TUNEL assay to reveal apoptosis of renal tissues. Scale bar, 50 μm. **(E)** Semi-quantification for renal tissue damage. **(F)** Statistical analysis for TUNEL-positive cells. All the data are shown as the mean ± *SD* (*n* = 6). *p < 0.05 vs. the relevant ND + I/R group.

According to previous results, compared with non-diabetic mice, RvD1 was significantly downregulated in STZ-induced diabetic mice. RvD1 treatment could ameliorate renal function and pathological injury after ischemic injury in STZ-induced diabetic mice. Based on these results, we believe that lower levels of RvD1 in diabetes may be a crucial factor in the increased susceptibility of diabetic patients to ischemic injury. To verify this conjecture, we treated C57 mice (no diabetes mice, ND) with BOC-2 (a selective RvD1 receptor inhibitor, 5 μg/kg) (Zhao et al., 2016; Pan et al., 2018). Firstly, we verified that BOC-2 treatment alone (without IR injury) would not cause any kidney injury in ND mice. In ND mice, 30 min of bilateral renal ischemia followed by I/R48 resulted in increases in BUN and creatinine. However, compared with the ND + I/R group, BOC-2 treatment could significantly deteriorate renal function when the mice suffered from ischemic injury (Figures 4A,B). HE staining also revealed dramatically more tissue damage in BOC-2-treated mice (Figures 4C,E). BOC-2 also significantly increased the apoptotic cells when the ND mice suffered from ischemic injury (Figures 4D,F). All of the data indicate that, even in non-diabetic mice, if we inhibit the effect of RvD1, the mice are also susceptible to ischemic AKI.

### RvD1 Inhibits NF-κB Activation After Ischemic Injury in STZ-Induced Diabetic Mice

**FIGURE 5:**
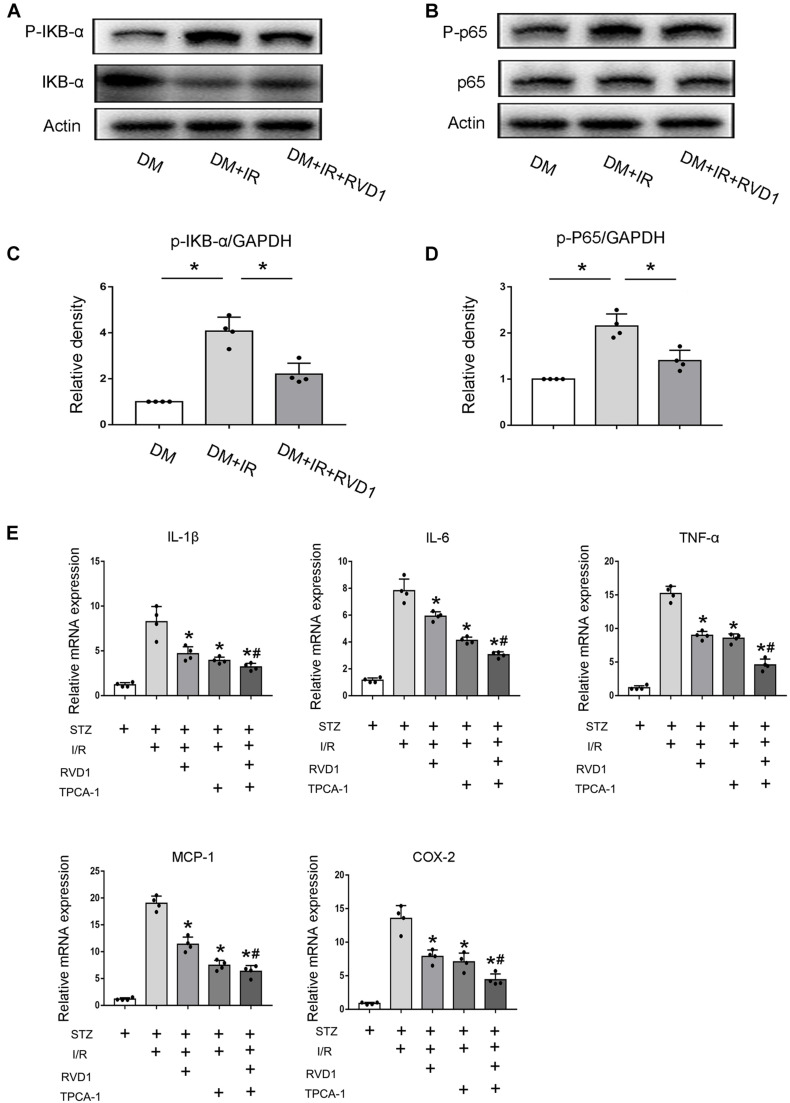
Resolvin D1 (RvD1) inhibits NF-κB activation after ischemic injury in streptozotocin (STZ)-induced diabetic mice. Male C57BL/6J mice were injected with STZ or vehicle solution to induce diabetic mice (DM) or non-diabetic mice (ND). The mice were then subjected to sham operation or 30 min of bilateral renal ischemia followed by 48 h of reperfusion (I/R48). For some groups, RvD1 (50 μg/kg) or TPCA-1 (5 mg/kg) was injected intraperitoneally 30 min before renal ischemia–reperfusion. Renal tissues were collected at 48 h of reperfusion for further analysis. **(A,B)** Immunoblot analysis for IKBα, p-IKBα, p65, and P-p65. GAPDH was used as the internal control. **(C,D)** Densitometry analysis of the p-p65 and p-IκB-α levels in kidney tissues. Data are shown as the mean ± *SD* (*n* = 4). *p < 0.05. **(E)** Real-time PCR for IL-1, IL-6, cTNF-α, MCP-1, and COX-2. Data are shown as the mean ± *SD* (*n* = 4). *p < 0.05 vs. the relevant STZ + I/R group; ^#^*p* < 0.05 vs. the relevant STZ + I/R + RvD1 group.

Just as described above, persistent activation of NF-κB would promote the release of inflammatory factors and enhance the inflammatory response, finally aggravating tissue damage. Our data indicated that ischemic injury led to the activation of NF-κB in the kidney tissue of diabetic mice, which was achieved by the significantly increased phosphorylation of IκBα. RvD1 treatment reduced IκBα phosphorylation (Figures 5A,C). Additionally, regarding p65, a crucial subunit of NF-κB, the phosphorylation of p65 was also upregulated. RvD1 treatment also reduced p65 phosphorylation (Figures 5B,D). Our data indicate that ischemic injury-induced NF-κB activation was inhibited by RvD1 treatment in diabetic mice. To verify the involvement of NF-κB signaling in RvD1 treatment following ischemic injury in diabetic mice, TPCA-1 (a most used NF-κB signaling pathway inhibitor) was applied in the experiment (5 mg/kg) (Podolin et al., 2005). The inflammatory factors IL-1β, IL-6, TNF-α, MCP-1, and COX-2 were detected through real-time PCR. Compared with treatment with RvD1 or TPCA-1 alone, a milder inflammatory response was observed when RvD1 or TPCA-1 was administered simultaneously. However, the synergism was incomplete, in as much as the combined effect seemed smaller than the sum of their individual action (Figure 5E).

## Discussion

This study indicated that RvD1 could attenuate susceptibility to ischemic AKI in diabetes. In addition, our study also verified that RvD1 attenuated susceptibility to ischemic AKI in diabetes mainly by downregulating NF-κB signaling and inhibiting apoptosis. Moreover, the present study suggested that patients with high RvD1 levels have better tolerance to ischemic AKI compared to patients with low RvD1 levels. Overall, these results suggested a novel strategy for RvD1 treatment of DKD, thereby providing a new therapy option.

Extensive evidence has verified that CKD is a crucial risk factor for the development of AKI. For example, James et al. conducted a meta-analysis in 2015 showing that CKD was a risk factor for developing AKI in patients with diabetes or hypertension (James et al., 2015). CKD was also shown to be an independent risk factor of AKI in patients with major cardiac surgery (Chung et al., 2016). In animal models, there is evidence that ischemic AKI is significantly more severe in diabetic mice than in non-diabetic mice (Peng et al., 2015). In our clinical results, all patients underwent surgery with cardiopulmonary bypass and aortic cross-clamping operations, mainly to simulate the renal ischemia–reperfusion environment in human patients. Within 7 days after surgery, the serum creatinine levels increased more relative to the baseline values in DKD patients compared with NDKD patients, and the incidence of AKI after surgery was 58.3% in the DKD group compared with 25% in the NDKD group. In our animal experiments, more renal damage and cell apoptosis occurred in the DM group. All the data are consistent with the evidence, indicating that CKD is more susceptible to ischemic AKI.

Despite extensive research focusing on AKI in CKD, the underlying mechanism is still poorly understood. The pathogenesis of AKI on CKD is multifactorial, involving chronic inflammation, vascular dysfunction, mitochondrial dysfunction, and oxidative stress (He et al., 2017). Multiple molecular mediators/regulators are responsible for the process, such as p53, hypoxia-inducible factor, and transforming growth factor beta (Kelly et al., 2003; Eckardt et al., 2005; Gewin and Zent, 2012; Cao et al., 2018). In this study, we mainly discussed the role of RvD1 in AKI on CKD. RvD1, one of the most studied RVs, has been reported to play a protective role in various animal disease models, including AKI, insulin resistance, peritonitis, wound infection, atherosclerosis, and I/R injury (Zhao et al., 2016). It has also been verified that RvD1 serves as a renal protective factor mainly through inhibiting inflammatory response (Cao et al., 2018; Wang et al., 2018).

RvD1 is a class of anti-inflammatory bioactive small molecules with conformational specificity that is derived from polyunsaturated fatty acids enzymatically in natural conditions. In order to generate RvD1, docosahexaenoic acid (DHA) is metabolized by both 5-LOX and 15-LOX, which selectively interacts with receptors ALX/FPR2 and GPR32 (Serhan et al., 2002; Krishnamoorthy et al., 2012). However, in diabetes or high-glucose culture conditions, the enzymatic levels of 15-LOX are decreased, finally resulting in decreased RvD1 levels in diabetes (Shi et al., 2017). In our study, we also found that the serum RvD1 level was downregulated in DM compared with ND mice. Our findings, combined with existing data, indicated the crucial role of RvD1 in AKI in CKD. To verify this, we treated DM mice with RvD1 by vein injection before the ischemic injury. The results indicated that RvD1 treatment could significantly improve the renal function and pathological damage, as well as reduce cell apoptosis when the mice suffered from ischemic injury. Based on these results, we believe that the lower levels of RvD1 in DM mice may be responsible for their increased susceptibility to ischemic injury. In order to further clarify this problem, we treated C57 mice (non-diabetic mice, ND) with BOC-2 (a selective RvD1 receptor inhibitor). As described above, the serum RvD1 levels were higher in ND than in DM mice. Therefore, BOC-2 was used to block the effects of RvD1 in ND mice. Conversely, BOC-2 treatment in ND mice could significantly aggravate the renal function and pathological damage, as well as increase cell apoptosis, when the mice suffered from ischemic injury. In clinical analysis, our results revealed that serum RvD1 levels were negatively associated with the creatinine elevation rate after surgery in DKD patients. In both DKD and NDKD participants, the creatinine elevation rates were higher in groups with low levels of RvD1 compared to those in groups with high levels of RvD1. All of the findings demonstrated that the downregulated serum RvD1 levels could be a crucial factor for susceptibility to ischemic AKI in diabetes.

It has been demonstrated that both tubular cell apoptosis and inflammatory response are related to susceptibility to ischemic AKI in diabetes (Peng et al., 2015; He et al., 2017). Generally, p53 is a major mediator of tubular cell apoptosis and death in AKI (Kelly et al., 2003). Peng et al. (2015) verified that p53 knockout from proximal tubules attenuates the sensitivity of STZ-induced diabetic mice to ischemic AKI, providing evidence that inhibiting cell apoptosis could attenuate the susceptibility to ischemic AKI in diabetes. The pro-inflammatory cytokines, which are upregulated in CKD, also play critical roles in the induction and progression of AKI (He et al., 2017). For example, TNF-α has been reported to increase the severity of ischemic AKI in diabetic mice (Gao et al., 2013). RvD1 has been shown to have anti-inflammatory and anti-apoptotic effects (Shao et al., 2016). Based on these data, we believe that RvD1 attenuates susceptibility to ischemic AKI in diabetes through inhibiting cell apoptosis and inflammatory response. In our study, RvD1 treatment significantly reduced the cell apoptosis and inflammatory response when the STZ-induced diabetic mice suffered from I/R injury, finally resulting in less renal structural and functional damage. These results indicated that RvD1 mainly attenuates susceptibility to ischemic AKI in diabetes through inhibiting cell apoptosis and inflammatory response.

NF-κB is a ubiquitously expressed transcription factor that regulates many inflammatory cytokines (Shao et al., 2016). Previous studies have reported that RvD1 markedly reduced acute lung injury-associated mortality by inhibiting the activation of the NF-κB pathway (Wang et al., 2011). In addition, RvD1 has been shown to regulate NF-κB activation to reduce mucosal inflammation (Colby et al., 2016). RvD1 has also been reported to inhibit the NF-κB signaling pathway and cytokines to protect against I/R kidney injury (Duffield et al., 2006). Based on this evidence, we believe that RvD1 may inhibit inflammatory response by targeting the NF-κB pathway. In our study, to verify the involvement of NF-κB signaling in RvD1 treatment following ischemic injury in diabetic mice, TPCA-1, a commonly used inhibitor of NF-κB signaling, was applied in the experiment. TPCA-1 is an IκB kinase inhibitor that blocks IκB phosphorylation and the consequent degradation, resulting in the accumulation of IκB to inhibit NF-κB (Podolin et al., 2005; Hao et al., 2017). Compared to treatment with RvD1 or TPCA-1 alone, a milder inflammatory response was observed when RvD1 or TPCA-1 was administered simultaneously. The results indicated that RvD1 and TPCA-1 might have a common action mechanism.

In summary, the present study suggested that RvD1 attenuated susceptibility to ischemic AKI in diabetes by downregulating NF-κB signaling and inhibiting apoptosis. Downregulated serum RvD1 levels could be the crucial factor for the susceptibility to ischemic AKI in diabetes. Overall, these results suggested a novel strategy for RvD1 treatment of AKI on CKD, thereby providing a new therapy option. However, as a promising therapeutic compound for AKI on CKD, further research is needed to shed light on its more detailed mechanism in anti-inflammatory and anti-apoptotic pathways.

## Data Availability Statement

The original contributions presented in the study are included in the article/supplementary material, further inquiries can be directed to the corresponding author/s.

## Ethics Statement

The studies involving human participants were reviewed and approved by the Ethics Committee of The Second Xiangya Hospital, Central South University. The patients/participants provided their written informed consent to participate in this study. The animal study was reviewed and approved by Ethics Committee of the Second Xiangya Hospital, Central South University.

## Author Contributions

LH designed the study. ZLi and ZLiu performed the experiments. HL and WD contributed to collection of human patient samples and clinical data. JC contributed to data analysis. ZLi, ZLiu, and LH contributed to data analysis and manuscript preparation. All authors contributed to the article and approved the submitted version.

## Conflict of Interest

The authors declare that the research was conducted in the absence of any commercial or financial relationships that could be construed as a potential conflict of interest.

## References

[B1] Bjork M. Dahlstrom O. Wettero J. Sjöwall C. (2015). Quality of life and acquired organ damage are intimately related to activity limitations in patients with systemic lupus erythematosus. *BMC Musculoskelet. Disord.* 16:188. 10.1186/s12891-015-0621-3 26264937 PMC4531389

[B2] Breyer M. D. Bottinger E. Brosius F. C. III Coffman T. M. Harris R. C. Heilig C. W. (2005). Mouse models of diabetic nephropathy. *J. Am. Soc. Nephrol.* 16 27–45. 15563560 10.1681/ASN.2004080648

[B3] Cao D. Pi J. Shan Y. Tang Y. Zhou P. (2018). Anti-inflammatory effect of Resolvin D1 on LPS-treated MG-63 cells. *Exp. Ther. Med.* 16 4283–4288. 30402165 10.3892/etm.2018.6721 PMC6201049

[B4] Chawla L. S. Eggers P. W. Star R. A. Kimmel P. L. (2014). Acute kidney injury and chronic kidney disease as interconnected syndromes. *N. Engl. J. Med.* 371 58–66. 24988558 10.1056/NEJMra1214243 PMC9720902

[B5] Chung C. U. Nelson J. A. Fischer J. P. Wink J. D. Serletti J. M. Kovach S. J. (2016). Acute kidney injury after open ventral hernia repair: an analysis of the 2005-2012 ACS-NSQIP datasets. *Hernia* 20 131–138. 10.1007/s10029-015-1395-0 26099501

[B6] Colby J. K. Abdulnour R. E. Sham H. P. Dalli J. Colas R. A. Winkler J. W. (2016). Resolvin D3 and aspirin-triggered resolvin D3 are protective for injured epithelia. *Am. J. Pathol.* 186 1801–1813. 10.1016/j.ajpath.2016.03.011 27171898 PMC4929400

[B7] Duffield J. S. Hong S. Vaidya V. S. Lu Y. Fredman G. Serhan C. N. (2006). Resolvin D series and protectin D1 mitigate acute kidney injury. *J. Immunol.* 177 5902–5911. 10.4049/jimmunol.177.9.5902 17056514

[B8] Eckardt K. U. Bernhardt W. M. Weidemann A. Warnecke C. Rosenberger C. Wiesener M. S. (2005). Role of hypoxia in the pathogenesis of renal disease. *Kidney Int. Suppl* 68 S46–S51. 10.1111/j.1523-1755.2005.09909.x 16336576

[B9] Gao G. Zhang B. Ramesh G. Betterly D. Tadagavadi R. K. Wang W. (2013). TNF-alpha mediates increased susceptibility to ischemic AKI in diabetes. *Am. J. Physiol. Renal Physiol.* 304 F515–F521. 23283990 10.1152/ajprenal.00533.2012 PMC3602710

[B10] Gewin L. Zent R. (2012). How does TGF-beta mediate tubulointerstitial fibrosis? *Semin. Nephrol.* 32 228–235. 10.1016/j.semnephrol.2012.04.001 22835453 PMC4948283

[B11] Hao J. Lou Q. Wei Q. Mei S. Li L. Wu G. (2017). MicroRNA-375 is induced in cisplatin nephrotoxicity to repress hepatocyte nuclear factor 1-beta. *J. Biol. Chem.* 292 4571–4582. 10.1074/jbc.m116.754929 28119452 PMC5377773

[B12] He L. Wei Q. Liu J. Yi M. Liu Y. Liu H. (2017). AKI on CKD: heightened injury, suppressed repair, and the underlying mechanisms. *Kidney Int.* 92 1071–1083. 10.1016/j.kint.2017.06.030 28890325 PMC5683166

[B13] James M. T. Grams M. E. Woodward M. Elley C. R. Green J. A. Wheeler D. C. (2015). A meta-analysis of the association of estimated GFR, albuminuria, diabetes mellitus, and hypertension with acute kidney injury. *Am. J. Kidney Dis.* 66 602–612. 25975964 10.1053/j.ajkd.2015.02.338 PMC4594211

[B14] Kanwar Y. S. Sun L. Xie P. Liu F. Y. Chen S. (2011). A glimpse of various pathogenetic mechanisms of diabetic nephropathy. *Annu. Rev. Pathol.* 6 395–423. 10.1146/annurev.pathol.4.110807.092150 21261520 PMC3700379

[B15] Kelly K. J. Plotkin Z. Vulgamott S. L. Dagher P. C. (2003). P53 mediates the apoptotic response to GTP depletion after renal ischemia-reperfusion: protective role of a p53 inhibitor. *J. Am. Soc. Nephrol.* 14 128–138. 10.1097/01.asn.0000040596.23073.01 12506145

[B16] Keyes K. T. Ye Y. Lin Y. Zhang C. Perez-Polo J. R. Gjorstrup P. (2010). Resolvin E1 protects the rat heart against reperfusion injury. *Am. J. Physiol. Heart Circ. Physiol.* 299 H153–H164. 20435846 10.1152/ajpheart.01057.2009

[B17] Krishnamoorthy S. Recchiuti A. Chiang N. Fredman G. Serhan C. N. (2012). Resolvin D1 receptor stereoselectivity and regulation of inflammation and proresolving microRNAs. *Am. J. Pathol.* 180 2018–2027. 10.1016/j.ajpath.2012.01.028 22449948 PMC3349829

[B18] Linkermann A. Chen G. Dong G. Kunzendorf U. Krautwald S. Dong Z. (2014). Regulated cell death in AKI. *J. Am. Soc. Nephrol.* 25 2689–2701. 10.1681/asn.2014030262 24925726 PMC4243360

[B19] Pan S. Wu Y. Pei L. Li S. Song L. Xia H. (2018). BML-111 reduces neuroinflammation and cognitive impairment in mice with sepsis via the SIRT1/NF-kappaB signaling pathway. *Front. Cell Neurosci.* 12:267. 10.3389/fncel.2018.00267 30186119 PMC6110933

[B20] Panera N. Della Corte C. Crudele A. Stronati L. Nobili V. Alisi A. (2016). Recent advances in understanding the role of adipocytokines during non-alcoholic fatty liver disease pathogenesis and their link with hepatokines. *Expert Rev. Gastroenterol. Hepatol.* 10 393–403. 10.1586/17474124.2016.1110485 26654761

[B21] Peng J. Li X. Zhang D. Chen J. K. Su Y. Smith S. B. (2015). Hyperglycemia, p53, and mitochondrial pathway of apoptosis are involved in the susceptibility of diabetic models to ischemic acute kidney injury. *Kidney Int.* 87 137–150. 10.1038/ki.2014.226 24963915 PMC4276728

[B22] Podolin P. L. Callahan J. F. Bolognese B. J. Li Y. H. Carlson K. Davis T. G. (2005). Attenuation of murine collagen-induced arthritis by a novel, potent, selective small molecule inhibitor of IkappaB Kinase 2, TPCA-1 (2-[(aminocarbonyl)amino]-5-(4-fluorophenyl)-3-thiophenecarboxamide), occurs via reduction of proinflammatory cytokines and antigen-induced T cell Proliferation. *J. Pharmacol. Exp. Ther.* 312 373–381. 10.1124/jpet.104.074484 15316093

[B23] Qu X. Zhang X. Yao J. Song J. Nikolic-Paterson D. J. Li J. (2012). Resolvins E1 and D1 inhibit interstitial fibrosis in the obstructed kidney via inhibition of local fibroblast proliferation. *J. Pathol.* 228 506–519. 10.1002/path.4050 22610993

[B24] Serhan C. N. Hong S. Gronert K. Colgan S. P. Devchand P. R. Mirick G. (2002). Resolvins: a family of bioactive products of omega-3 fatty acid transformation circuits initiated by aspirin treatment that counter proinflammation signals. *J. Exp. Med.* 196 1025–1037. 12391014 10.1084/jem.20020760 PMC2194036

[B25] Shao A. Wu H. Hong Y. Tu S. Sun X. Wu Q. (2016). Hydrogen-rich saline attenuated subarachnoid hemorrhage-induced early brain injury in rats by suppressing inflammatory response: possible involvement of NF-kappaB pathway and NLRP3 inflammasome. *Mol. Neurobiol.* 53 3462–3476. 10.1007/s12035-015-9242-y 26091790

[B26] Shi H. Carion T. W. Jiang Y. Chahine A. Steinle J. J. Berger E. A. (2017). A regulatory role for beta-adrenergic receptors regarding the resolvin D1 (RvD1) pathway in the diabetic retina. *PLoS One* 12:e0185383. 10.1371/journal.pone.0185383 29095817 PMC5667888

[B27] Tang C. Han H. Liu Z. Liu Y. Yin L. Cai J. (2019). Activation of BNIP3-mediated mitophagy protects against renal ischemia-reperfusion injury. *Cell Death Dis.* 10:677. 31515472 10.1038/s41419-019-1899-0 PMC6742651

[B28] Tang S. C. W. Yiu W. H. (2020). Innate immunity in diabetic kidney disease. *Nat. Rev. Nephrol.* 16 206–222. 10.1038/s41581-019-0234-4 31942046

[B29] Venkatachalam M. A. Griffin K. A. Lan R. Geng H. Saikumar P. Bidani A. K. (2010). Acute kidney injury: a springboard for progression in chronic kidney disease. *Am. J. Physiol. Renal Physiol.* 298 F1078–F1094. 10.1016/j.ymed.2011.08.050 20200097 PMC2867413

[B30] Venkatachalam M. A. Weinberg J. M. Kriz W. Bidani A. K. (2015). Failed tubule recovery, AKI-CKD transition, and kidney disease progression. *J. Am. Soc. Nephrol.* 26 1765–1776. 10.1681/asn.2015010006 25810494 PMC4520181

[B31] Wang B. Gong X. Wan J. Y. Zhang L. Zhang Z. Li H. Z. (2011). Resolvin D1 protects mice from LPS-induced acute lung injury. *Pulm. Pharmacol. Ther.* 24 434–441. 10.1016/j.pupt.2011.04.001 21501693

[B32] Wang X. Jiao W. Lin M. Lu C. Liu C. Wang Y. (2018). Resolution of inflammation in neuromyelitis optica spectrum disorders. *Mult. Scler. Relat. Disord.* 27 34–41. 30300851 10.1016/j.msard.2018.09.040

[B33] Wei Q. Dong Z. (2012). Mouse model of ischemic acute kidney injury: technical notes and tricks. *Am. J. Physiol. Renal Physiol.* 303 F1487–F1494. 22993069 10.1152/ajprenal.00352.2012 PMC3532486

[B34] Xu J. Duan X. Hu F. Poorun D. Liu X. Wang X. (2018). Resolvin D1 attenuates imiquimod-induced mice psoriasiform dermatitis through MAPKs and NF-kappaB pathways. *J. Dermatol. Sci.* 89 127–135. 10.1016/j.jdermsci.2017.10.016 29137840

[B35] Yang S. Zhao L. Han Y. Liu Y. Chen C. Zhan M. (2017). Probucol ameliorates renal injury in diabetic nephropathy by inhibiting the expression of the redox enzyme p66Shc. *Redox Biol.* 13 482–497. 10.1016/j.redox.2017.07.002 28728079 PMC5514499

[B36] Yin Y. Chen F. Wang W. Wang H. Zhang X. (2017). Resolvin D1 inhibits inflammatory response in STZ-induced diabetic retinopathy rats: possible involvement of NLRP3 inflammasome and NF-kappaB signaling pathway. *Mol. Vis.* 23 242–250. 28465656 PMC5398882

[B37] Zhang X. Qu X. Sun Y. B. Caruana G. Bertram J. F. Nikolic-Paterson D. J. (2013). Resolvin D1 protects podocytes in adriamycin-induced nephropathy through modulation of 14-3-3beta acetylation. *PLoS One* 8:e67471. 10.1371/journal.pone.0067471 23840712 PMC3696081

[B38] Zhao Y. L. Zhang L. Yang Y. Y. Tang Y. Zhou J. J. Feng Y. Y. (2016). Resolvin D1 protects lipopolysaccharide-induced acute kidney injury by down-regulating nuclear factor-kappa B signal and inhibiting apoptosis. *Chin. Med. J. (Engl.)* 129 1100–1107. 10.4103/0366-6999.180517 27098797 PMC4852679

